# Technical Reproducibility of Genotyping SNP Arrays Used in Genome-Wide Association Studies

**DOI:** 10.1371/journal.pone.0044483

**Published:** 2012-09-07

**Authors:** Huixiao Hong, Lei Xu, Jie Liu, Wendell D. Jones, Zhenqiang Su, Baitang Ning, Roger Perkins, Weigong Ge, Kelci Miclaus, Li Zhang, Kyunghee Park, Bridgett Green, Tao Han, Hong Fang, Christophe G. Lambert, Silvia C. Vega, Simon M. Lin, Nadereh Jafari, Wendy Czika, Russell D. Wolfinger, Federico Goodsaid, Weida Tong, Leming Shi

**Affiliations:** 1 Center of Excellence for Bioinformatics, Division of Systems Biology, National Center for Toxicological Research, U.S. Food and Drug Administration, Jefferson, Arizona, United States of America; 2 Division of Personalized Nutrition and Medicine, National Center for Toxicological Research, U.S. Food and Drug Administration, Jefferson, Arizona, United States of America; 3 Expression Analysis Inc., Durham, North Carolina, United States of America; 4 ICF International Company at National Center for Toxicological Research, U.S. Food and Drug Administration, Jefferson, Arizona, United States of America; 5 SAS Institute Inc, Cary, North Carolina, United States of America; 6 Office of Clinical Pharmacology, Center for Drug Evaluation and Research, U.S. Food and Drug Administration, Silver Spring, Maryland, United States of America; 7 Samsung Advanced Institute of Technology, Giheung-gu, Yongin-si Gyeonggi-do, Republic of Korea; 8 Center of Excellence for Genomics, Division of Systems Biology, National Center for Toxicological Research, U.S. Food and Drug Administration, Jefferson, Arizona, United States of America; 9 Golden Helix Inc., Bozeman, Montana, United States of America; 10 Rosetta BioSoftware, Health Solutions Group, Microsoft, Seattle, Washington, United States of America; 11 Biomedical Informatics Research Center, Marshfield Clinic Research Foundation, Marshfield, Wisconsin, United States of America; 12 Center for Genetic Medicine, Northwestern University, Chicago, Illinois, United States of America; Auburn University, United States of America

## Abstract

During the last several years, high-density genotyping SNP arrays have facilitated genome-wide association studies (GWAS) that successfully identified common genetic variants associated with a variety of phenotypes. However, each of the identified genetic variants only explains a very small fraction of the underlying genetic contribution to the studied phenotypic trait. Moreover, discordance observed in results between independent GWAS indicates the potential for Type I and II errors. High reliability of genotyping technology is needed to have confidence in using SNP data and interpreting GWAS results. Therefore, reproducibility of two widely genotyping technology platforms from Affymetrix and Illumina was assessed by analyzing four technical replicates from each of the six individuals in five laboratories. Genotype concordance of 99.40% to 99.87% within a laboratory for the sample platform, 98.59% to 99.86% across laboratories for the same platform, and 98.80% across genotyping platforms was observed. Moreover, arrays with low quality data were detected when comparing genotyping data from technical replicates, but they could not be detected according to venders’ quality control (QC) suggestions. Our results demonstrated the technical reliability of currently available genotyping platforms but also indicated the importance of incorporating some technical replicates for genotyping QC in order to improve the reliability of GWAS results. The impact of discordant genotypes on association analysis results was simulated and could explain, at least in part, the irreproducibility of some GWAS findings when the effect size (i.e. the odds ratio) and the minor allele frequencies are low.

## Introduction

The International HapMap Project determined genotypes of over 3.1 million common SNPs in human populations [1]. Concurrent advancement in high-throughput SNP genotyping technology enabled simultaneous genotyping of hundreds of thousands of SNPs, making GWAS a feasible and a promising research field for associating genotypes with various disease susceptibilities and health outcomes. Common genetic variants associated with the risk of more than 200 diseases and human phenotypic traits have been identified using GWAS [2]–[26] (http://www.genome.gov/gwastudies/). However, most genetic markers identified with GWAS confer very small relative risks, usually with odds ratios between 1.1 to 1.5, even though the identified markers met a very stringent statistical significance criterion (i.e., a very small *p* value, usually as a result of large sample sizes) [2]. Moreover, replication studies demonstrate that only a small portion of associated loci in the initial GWAS can be replicated, even within the same populations. For example, in replication studies of GWAS for type 2 diabetes mellitus, Zeggeni *et al.* [3] replicated associations for only ten out of 77 SNP-based loci tested, Scott *et al.* [4] ten of 80, Easton *et al.* [5] eight of 57, and Steinthorsdottir *et al.* [6] two of 47. SNP lists identified in different GWAS for the same disease tend to be quite disparate. For instance, among the four confirmed SNPs associated with type 2 diabetes by Rung *et al.* [7], only rs7903146 in gene TCF7L2 was identified in previous GWAS, such as the GWAS by the Wellcome Trust Case Control Consortium [8]. Concerns have been raised recently regarding reliability and utility of GWAS findings [27], [28].

Given the complexity of GWAS, multiple potential sources of Type I and II errors exist. GWAS are based on the common trait-common variant hypothesis that implies genetic architecture of complex traits consists of a number of common alleles, each conferring a small increase in risk to the individual [29]. Since the likelihood of detecting an individual SNP association is usually small, a large sample size is needed to achieve adequate statistical power to detect true associations. The potential sources of Type I and II errors in GWAS include, but are not limited to, case-control misclassification [30], non-genetic covariates (e.g., smoking [31] and obesity [32]), and population stratification [33]. In addition, inaccurate genotyping data also affect the list of identified SNPs [34], [35]. Thus, efforts to detect, prevent, and eradicate sources of technical errors and biases in genotyping are important for improving the quality of genotype data and gaining confidence in GWAS results.

We previously examined the reproducibility of one platform by assessing the consistency in genotypes between technical replicates of six subjects assayed with the Affymetrix SNP array 6.0 (called Affy6 thereafter) in the same laboratory [36]. A reasonable level of intra-laboratory and intra-platform reproducibility was observed. However, reproducibility or genotype concordance between genotyping platforms and across laboratories has not been systematically evaluated. Therefore, this study was designed to evaluate the technical robustness of genotyping platforms and to further assess whether technical variability in genotyping is a potential source for the discordant findings in GWAS. Specifically, inter-laboratory and inter-platform reproducibility of genotypes was evaluated through comparisons of genotyping results of four technical replicates for six subjects across two different SNP arrays, Affy6 and Illumina 1MDuo chip (called Illu1M thereafter), in five different laboratories.

## Materials and Methods

### Ethics Statement

All DNA samples were obtained from other institutes and are anonymized and publically available. Therefore, subjects can not be identified either directly or through identifiers. Research on the samples is exempt from US FDA RIHSC review.

### DNA Samples

DNA samples for the three HapMap subjects (NA10385, NA12449, and NA12448, coded as A, B, and C in Table 1) are a HapMap trio and were obtained from Coriell Institute for Medical Research (Camden, NJ).

**Table 1:** Genotyping platforms and DNA samples and data analyzed in this study.

Genotyping Site*		1	2		3		4
Experiment ID		E1	E2		E3	E4	E5
Manufacturer		Affymetrix				Illumina	
Platform		Human SNP Array 6.0				Human1M-Duo	
Sample	Code	Data used					
HapMap NA10835	A	4		4	4	4	4
HapMap NA12249	B	4		2	4	4	4
HapMap NA12248	C	4		3	4	4	4
NCTR59	D	4		4	4	4	4
NCTR8	E	4		3	4	4	4
NCTR13	F	3		3	4	4	4
Total		23		19	24	24	24

* Genotyping site: 1: CoGenics; 2: Center for Molecular Medicine; 3: Express in Analysis; 4: Northwestern University.

The DNA samples of subjects NCTR59, NCTR8, and NCTR13 (coded as D, E, and F, respectively, in Table 1) are from three anonymous human liver specimens that were obtained from the US Cooperative Human Tissue Network (CHTN) that were used for human genomic DNA extraction, and these liver tissue samples were confirmed by a pathologist to be obtained from normal donors.

### HapMap Data

The three HapMap subjects have been genotyped by HapMap project using different genotyping platforms, including the Affymetrix GenomeWide 6.0 arrays and the Illumina 1MDuo chips that are used in this article. To compare our results with the HapMap results, the raw genotyping data of the three samples in HapMap project were obtained from Affymetrix and Illumina. The same genotype calling processes were applied to the HapMap data.

### Genotyping

Four replicates of DNA samples of the six subjects were genotyped using both Affy6 and Illu1M platforms according to the standard protocols. On a 96-well plate, DNA samples were placed in 24 wells. Each well contains 2.0∼2.5 µg of DNA at a concentration of ∼100 ng/µl. The 24 DNA samples were placed in three columns of the 96-well plate (samples are randomized on the plate in the layout depicted in Figure S1) for genotyping. Five such plates with the identical sample layout were prepared at NCTR and shipped to the five genotyping experiments such that sample information was blinded for genotyping.

SNP genotyping was performed with the commercial release of the Affymetrix GenomeWide 6.0 arrays at genotyping experiments E1, E2, and E3. In brief, 500 ng of DNA each was digested with NspI and StyI restriction enzymes, an adaptor was ligated and molecules were then fragmented and labeled. A generic PCR primer that recognizes the adaptor sequence was used to amplify adaptor-ligated DNA fragments from both restriction digests. The PCR conditions were optimized to preferentially amplify fragments in the 200 to 1,100 bp size range. At this stage the preparation was hybridized to the SNP array (906,600 SNPs). DAT images were interpreted using AGCC software and Genotyping Console.

SNP genotyping was performed with the commercial release of the Illumina 1MDuo chips at genotyping experiments E4 and E5. The assays began with 400 ng of DNA per sample and included an Illumina proprietary whole genomic amplification step followed by a fragmentation and precipitation of the DNA. The DNA was then resuspended and hybridized to the 1M-Duo chips (>1 million SNPs). The SNP was interrogated on the chip itself with a DNA extension step and the addition of the fluorescent marker.

### Genotype Calling

For the Affy6 platform, the quality of raw data was assessed using the program apt-geno-qc in the APT before genotype calling. Genotype calling was conducted using Birdseed version-1 through function apt-probeset-genotype in APT. All the parameters were set to the default values recommended by Affymetrix. In previous work, we assessed calling batch effect and found that uniform and large batch sizes with homogenous samples should be used to make genotype calls for GWAS^37^. Therefore, for this work, all of raw data of the 24 samples from one genotyping experiment were called in one batch.

For platform Illu1M, the raw intensity data from genotyping experiments E4 and E5 were genotype called separately. Genotype calling was conducted using the genotyping module v3.3.7 in the BeadStudio v3.1 (Illumina, San Diego, CA, USA). All the parameters were set to the default values recommended by Illumina. The manifest file (.bpm) and the cluster file (.egt) for Humna1M-Duo, which were downloaded from Illumina website, were used in genotype calling.

### Comparing Genotype Calling Results

The pair-wise concordances of genotypes between samples (replicates of the same subject) were calculated using the formula:
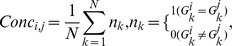
where *N* indicates total SNPs, 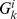 is the genotype called on SNP k for sample i, and 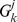
is the genotype called on SNP k for sample j.

### Simulations of the Impact of Genotyping Error on GWAS Associations

Simulations were conducted to estimate the effects of discordance in genotypes on the associated SNPs identified in GWAS. First, a data set with a case population of 2,000 samples and a control population of 3,000 samples was generated in which the same minor allele frequency was fixed and separately applied to the case and control populations. Thus, the original data set represented an odds ratio of exactly one. Thereafter, a fixed concordant rate in genotypes was randomly applied to the data set and an odds ratio was calculated on the simulated data. The process was repeated 50,000 times for a pair of fixed minor allele frequency and concordant rate. Therefore, 50,000 odds ratio values were obtained for a pair of minor allele frequency and concordant rate. Then, minor allele frequency and concordant rate were changed from 0 to 0.4 and from 1.00 to 0.94 with steps of 0.01 and −0.001, respectively. In a similar way, 50,000 odds ratios were simulated for each combination of minor allele frequency and concordance rate.

## Results

### Experimental Design and Data Generated and Used

To assess inter-laboratory and inter-platform reproducibility of genotyping technologies, DNA samples of three HapMap subjects and three US Cooperative Human Tissue Network subjects were prepared, each with four replicates. The 24 DNA samples were placed in three columns of five 96-well plates, and with placement randomized as shown in Figure S1 prior to shipping to the genotyping laboratories. Genotyping experiments were done in three different laboratories using Affy6 and in two different laboratories using Illu1M. After excluding the data of low quality, as discussed in the section “Replicate samples ensuring QC in GWAS”, the 114 samples listed in Table 1 were analyzed.

Birdseed-v1 in Affymetrix Power Tools (APT) (1.10.0) and the genotyping module v3.3.7 in Illumina BeadStudio v3.1 were used to make genotype calls for the 66 raw data files from Aff6 and the 48 raw data files from Illu1M, respectively.

The QC scores of the 66 CEL files from Affy6 were in the range of 88.6%–99.1% with an average score of 95.9% and a standard deviation of 3.1% (Figure S2) which are within Affymetrix guidelines. The 10% GC scores and the call rates of the 48 intensity files from Illu1M were in the ranges of 0.668–0.684 and 99.3%–100% (Figure S3), respectively, compliant with Illumina guidelines. Thus, the raw data were of acceptable genotyping quality for the comparative study.

The technical performance of genotyping platforms was assessed in terms of three types of reproducibility comparisons: 1) within a platform and within a laboratory; 2) across laboratories for individual platforms; and 3) across both platforms and laboratories.

### Intra-platform and Intra-laboratory Genotyping Reproducibility

**Figure 1:**
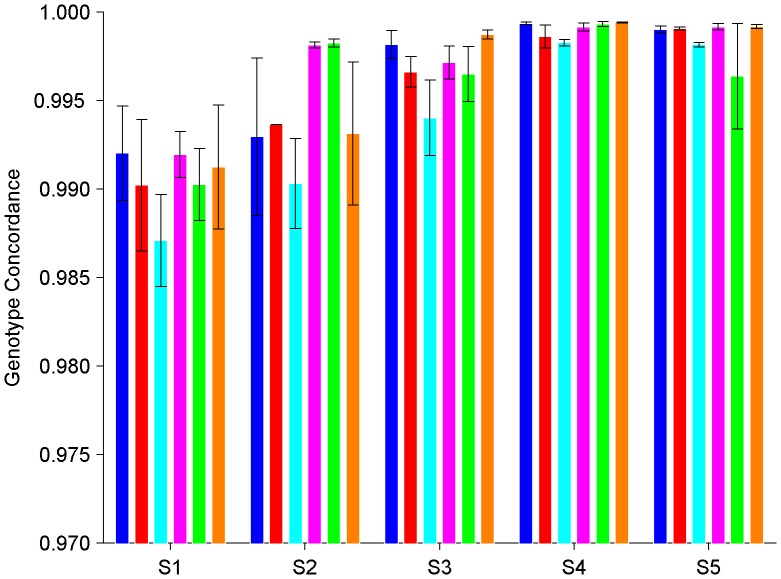
Concordance in genotypes between replicates of the same subject within a genotyping platform and within a genotyping experiment. The averaged concordance values (the bars) and the corresponding standard deviations (the error bars) between replicates of a subject (coded by color as: blue for A, red for B, cyan for C, Magenta for D, Green for E, and Orange for F) genotyped in a genotyping experiment (indicated at x-axis) are plotted. The subject codes and the experiment ID for genotyping experiments are listed in Table 1.

To measure genotyping reproducibility, genotype concordances were calculated for all pair-wise comparisons between the technical replicates of the DNA samples from six subjects for each genotyping experiment. Heatmaps of results for genotyping experiments E1 through E5 are shown in Figures S4, S5, S6, S7, S8, respectively. The concordances between technical replicates of DNA samples from the same subject within a genotyping platform and within a genotyping laboratory were high; greater than 99%. The averaged intra-platform and intra-laboratory genotype concordances as well as the corresponding standard deviations for the five genotyping experiments and six subjects are shown in Figure 1. Concordances for Affy6 were 99.04%, 99.48%, and 99.69% for genotyping experiments E1, E2 and E3, respectively, for an average of 99.40% with a standard deviation of 0.29%. Concordances for Illu1M were 99.90% and 99.85% for genotyping experiments E4 and E5, respectively, for an average of 99.87% with a standard deviation of 0.10%. Therefore, high intra-platform and intra-laboratory genotyping reproducibilities were observed by using technical replicates.

### Intra-platform and Inter-laboratory Genotyping Reproducibility

**Figure 2:**
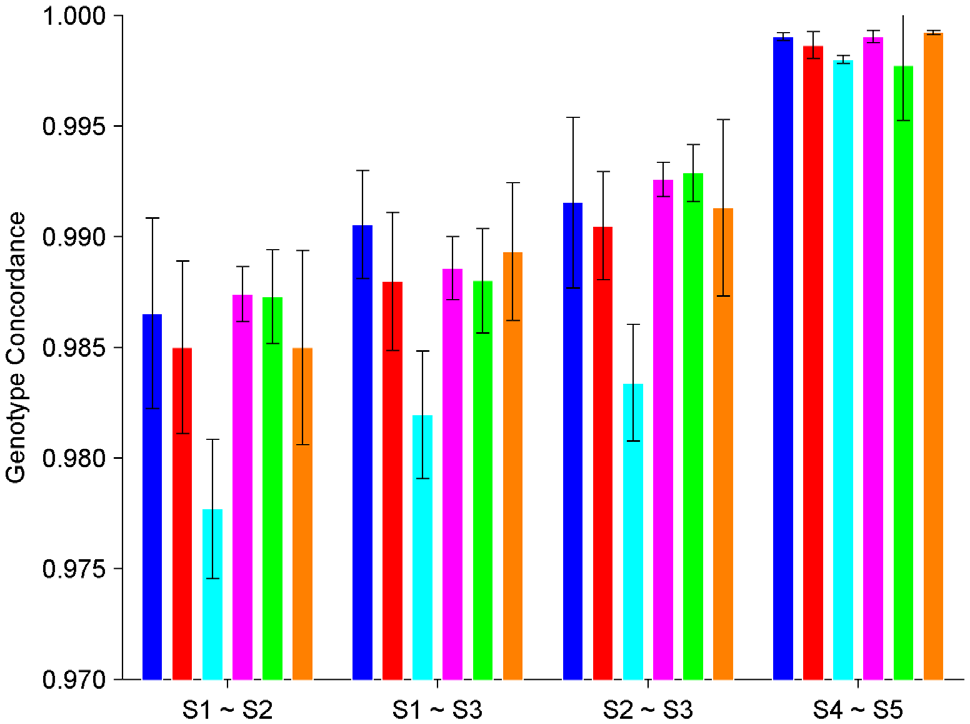
Concordance in genotypes between replicates of the same subject genotyped in two different experiments by using the same genotyping platform. The averaged concordance values (the bars) and the corresponding standard deviations (the error bars) between replicates of a subject (coded by color as: blue for A, red for B, cyan for C, Magenta for D, Green for E, and Orange for F) genotyped at two genotyping experiments (indicated at x-axis) are plotted. The subject codes and the experiment ID for genotyping experiments are listed in Table 1.

To determine the reproducibility between laboratories using the same platform, genotype calls were compared using data from the technical replicates genotyped using a same genotyping platform in different laboratories. Genotypes determined from SNPs of technical replicates of same subjects were compared for the Affy6 platform between experiments E1, E2, and E3, and separately for Illu1M platform between experiments E4 and E5. Genotype concordances were calculated for all of the pair-wise cross-experiment comparisons. Heatmaps of results for Affy6 cross-experiment comparisons are given in Figures S9, S10, S11 and for the Illu1M cross-experiment comparison in Figure S12. All concordances between technical replicates of DNA samples from the same subject genotyped in different laboratories using either Affy6 or Illu1M were greater than 98%. The averaged intra-platform and inter-laboratory genotype concordances as well as the corresponding standard deviations for the six subjects are shown in Figure 2. The overall averaged concordance for platform Affy6 was 98.59% (98.50%, 98.77%, and 98.50% for between experiments E1 and E2, between experiments E1 and E3, and between experiments E2 and E3, respectively) with a standard deviation of 0.43%, and for platform Illu1M was 99.86% (between experiments E4 and E5) with a standard deviation of 0.12%. Thus, a high intra-platform and inter-laboratory genotyping reproducibility was observed for both Affy6 and Illu1M platforms.

Genotypes of SNPs of the three HapMap subjects have been determined by the HapMap project using different genotyping platforms, including Affy6 and Illu1M that were used in this study. Albeit the genotyping reproducibility between laboratories in our study was evaluated, it is vital to assess the reliability of the data generated from this study. Therefore, we examined the consistency of the SNP calling results between our experiments and HapMap project data. Heatmaps of concordances of genotypes between our experiments and Hapmap project data for Affy6 and Illu1M are given in Figure S25 and Figure S26, respectively. The overall averaged concordance for platform Affy6 was 98.34% (98.01% between experiment E1 and HapMap, 98.07% between experiment E2 and HapMap, and 98.87% between experiment E3 and HapMap, respectively). The overall averaged concordance for platform Illu1M was 98.85% (99.87% between experiment E4 and HapMap and 99.83% between experiment E5 and HapMap, respectively). The results demonstrate that the data from our experiments are consistent with the data from HapMap project and thus the reproducibility reported in this paper has no biases.

### Inter-platform and Inter-laboratory Genotyping Reproducibility

**Figure 3:**
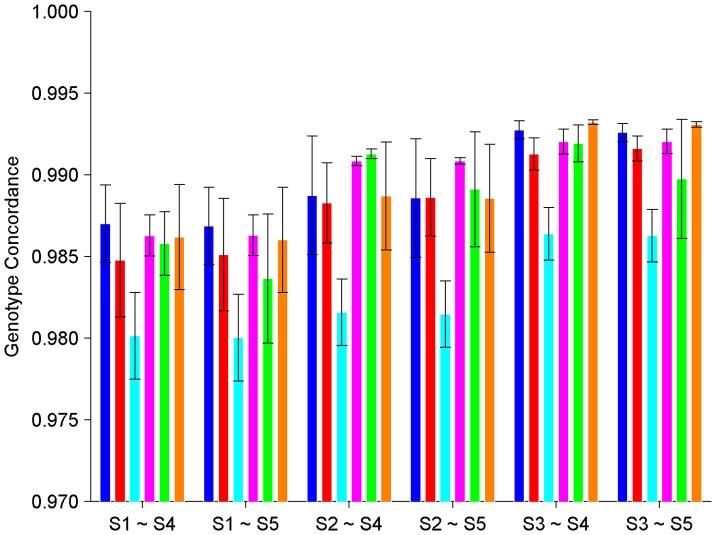
Concordance in genotypes between replicates of the same subject genotyped by using different genotyping platforms. The averaged concordance values (the bars) and the corresponding standard deviations (the error bars) between replicates of a subject (coded by color as: blue for A, red for B, cyan for C, Magenta for D, Green for E, and Orange for F) genotyped by using different genotyping platforms (indicated at x-axis) are plotted. The subject codes and the experiment ID for genotyping experiments are listed in Table 1.

Genotype concordances were calculated for all of the pair-wise comparisons between technical replicates of the DNA samples from the six subjects between platforms Affy6 and Illu1M. Heatmaps of the concordances are given in Figures S13, S14, S15, S16, S17, S18. The averaged inter-platform and inter-laboratory genotype concordances as well as the corresponding standard deviations for the six subjects are given in Figure 3. The overall averaged genotype concordance between platforms Affy6 and Illu1M was 98.80% with a standard deviation of 0.34%. Our data demonstrated high inter-platform and inter-laboratory genotyping reproducibility between genotyping platforms Affy6 and Illu1M.

### Replicate Samples Ensuring QC in GWAS

Genotyping QC is very important to mitigate false positive associations between genotypes and phenotypes. Vendors of genotyping platforms accordingly provide guidelines for genotyping QC to ensure high quality of data for subsequent association analysis. Our results show that QC in accordance with venders’ guidelines is necessary but might not be sufficient to assure data of adequate quality. The data from the 24 DNA samples of six subjects genotyped by platform Affy6 from genotyping experiment E1 met the QC criteria according to Affymetrix’s suggestions. However, when comparing genotype calling results between technical replicates of the same subjects it was observed that among the four technical replicates of subject F (NCTR13), one had a very disparate heterozygosity genotype rate compared with other replicates (Figure S19). The data of that particular replicate were deemed suspicious and thus excluded from the reproducibility analysis. At genotyping experiment E2, QC of one replicate of subject B (HapMap NA12249) on spot H3 (Figure S1) indicated low quality data and re-scanning the array and re-genotyping the sample did not correct the problem. The SNP arrays of the rest 23 DNA samples of the six subjects met the QC criteria according to Affymetrix’s guideline. In fact, one replicate from each of subjects B, C, E, and F had much higher heterozygous rate than other corresponding subject replicates (Figure S20); the quality of data for those six samples (one sample from experiment E1 and five samples from experiment E2) were deemed too low and excluded from the comparative study.

**Figure 4:**
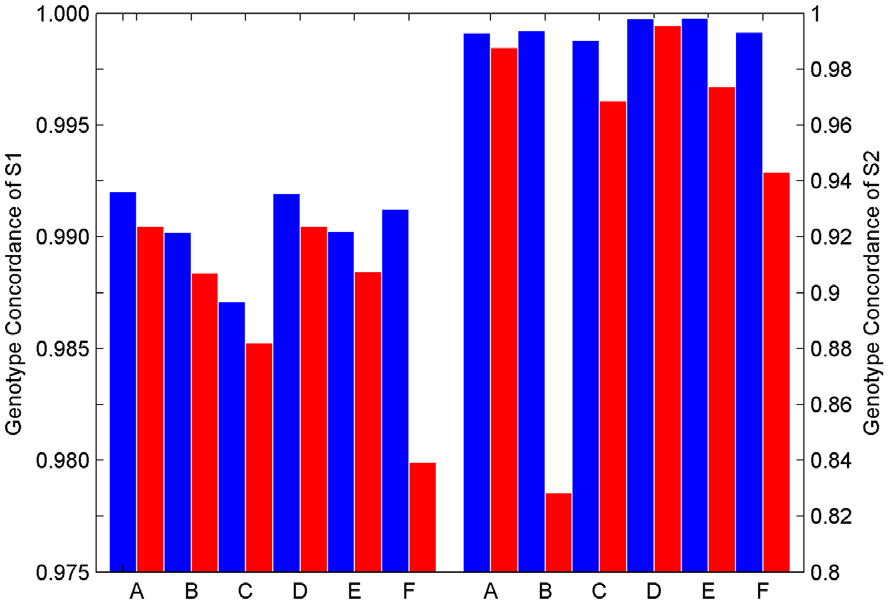
Concordance in genotypes between replicates of the same subject within a genotyping platform and within a genotyping experiment for comparing with (blue bars) and without (red bars) removal of arrays of low quality. The left panel is plotted for the data from genotyping experiment E1 while the right panel for genotyping experiment E2. The subject codes of the x-axis are listed in Table 1.

The comparative analyses were repeated after genotype calling by adding previously excluded low quality data back. Genotype concordances were calculated for all of the pair-wise comparisons between technical replicates of the six subjects for experiment E1 (Figure S21) and experiment E2 (Figure S22). The results are summarized and compared with the corresponding results without low quality data in Figure 4. Concordance of genotypes between replicates of a subject decreased when the low quality data were included in the data analysis. Moreover, inclusion of the low quality data not only decreased the intra-platform and intra-laboratory genotype reproducibility of the subjects with replicates of low quality (F of experiment E1; B, C, E, and F of experiment E2) but also affected the subjects without replicates of low quality.

### Discordant Genotypes Affect GWAS Findings

**Figure 5:**
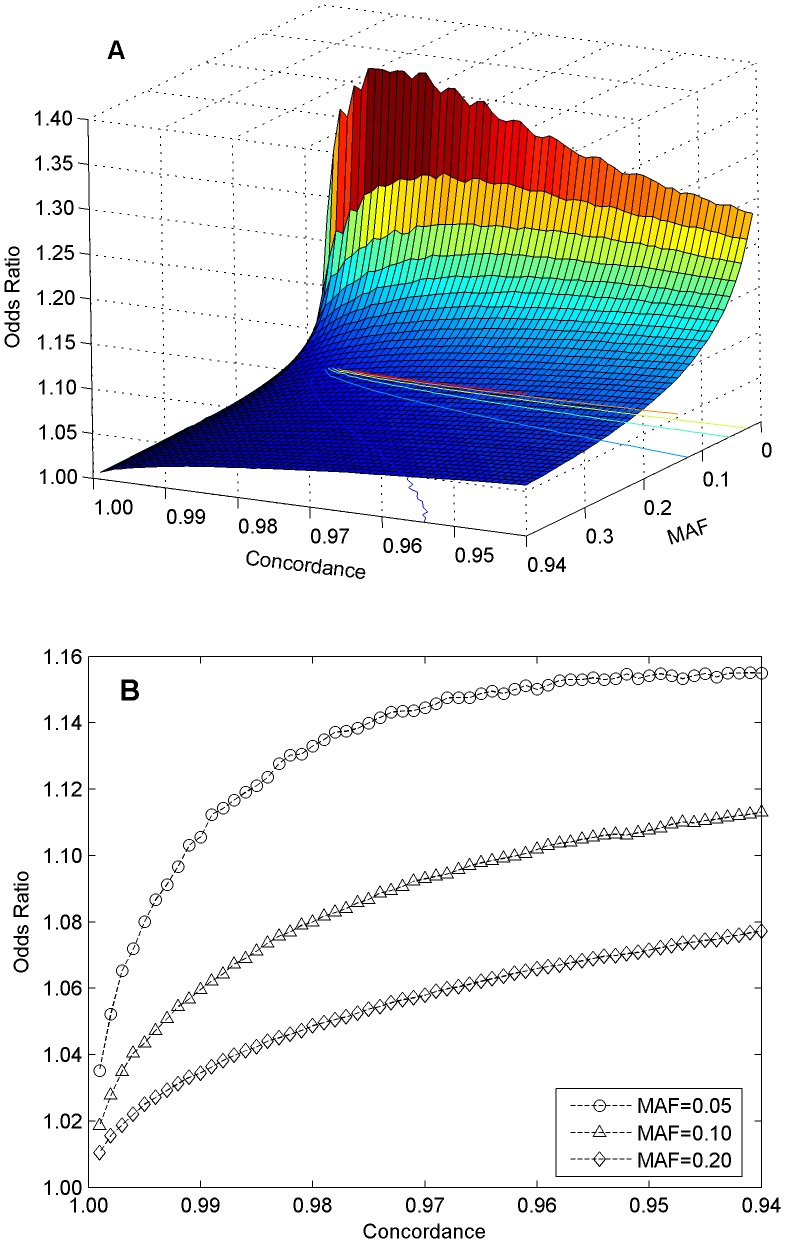
Simulations results. Odds ratios were simulated for 50,000 times for each pair of a genotype concordance (from 0.94 to 1.00 with a step of 0.001) and a minor allele frequency (from 0.01 to 0.40 with a step of 0.01) by using a case population of 2,000 samples and a control sample size of 3,000 samples. Relationship between top 5% odds ratio of the 50,000 ones, concordance in genotypes, and minor allele frequency is depicted in **A**. The intersection curves at minor allele frequency values 0.05, 0.10, and 0.20 are shown in **B**.

Reproducibility of 99.40%–99.87% within a laboratory, 98.59%–99.86% across laboratories, and 98.80% across genotyping platforms was observed by genotyping replicates in this study. Therefore, genotyping technologies could be considered robust and reproducible in terms of genotypes determined. However, most genetic markers identified in GWAS confer very small relative risks. Thus, a very small error in genotypes could be inflated in GWAS and might generate false associations. To assess the effect of discordant genotypes on the associated SNPs identified in GWAS, simulations were conducted by varying the minor allele frequency and the genotyping concordance rate. Figure 5 showed the results of simulations with a control population of 3,000 samples and a case population of 2,000 samples. In the simulations, at the same minor allele frequency for both control population and case population, the same concordance (or discordance) in genotypes was randomly applied to the simulated populations for 50,000 times. Therefore, 50,000 odds ratio values were obtained for a pair of fixed minor allele frequency and genotype concordance. Those odds ratios were not caused by differences in minor allele frequencies for control and case populations but were caused by the simulated variations in genotypes. Figure 5A gave the top five percentile values of the odds ratios and Figure 5B depicted the relationship between five percentile odds ratio and concordance in genotypes for some fixed minor allele frequency values. The trend is obvious in that the smaller the minor allele frequency and the lower the concordance in genotypes (measurement of reproducibility of genotyping), the larger the spurious (simulated) odds ratio. Therefore, our results revealed that a very small discordance in genotypes caused in genotyping could change odds ratios of genetic markers and affect the final conclusions of GWAS.

## Discussion

GWAS simultaneously interrogate hundreds of thousands of SNPs to search for genetic variants associated with health-related traits. In the past several years, many putative loci have been so identified and replicated [2]–[26]. Unfortunately, replication is often not included in GWAS protocols, raising the risk of inducing both Type I and II errors in different steps of a complicated process. Batch effects in genotype calling have been identified as a potential source of inferential errors in GWAS [37]–[41]. Variations among genotype calling algorithms and SNP arrays have also been observed to affect findings in GWAS [36]. Intrinsic limitations in genotyping technology could also be a potential source of Type I and II errors in GWAS. Therefore, it is important to understand the robustness of current genotyping technology used in GWAS, as assessed here.

Reasonable reproducibility, measured as concordance of genotypes between technical replicates of the same subject, both within and across genotyping experiments and both within and across genotyping platforms, was observed. Common diseases investigated in GWAS are typically influenced by multiple loci, with each locus making a small contribution to the overall risks. Therefore, small errors in any procedure in GWAS could be amplified in GWAS results, as demonstrated in the results of simulations (Figure 5) and our previous studies [35]–[37]. This study has shown that technical replicates enable low quality data to be identified and removed before corrupting genotyping results. Specifically, one replicate for subject F from genotyping experiment E1 (Figure S19) and one replicate for each of the subjects B, C, E, and F from genotyping experiment E2 (Figure S20) would not have been identified as low quality by vendors’ QC guidelines, leading to low concordance in genotypes. In contrast, the available technical replicates for the same subjects allowed disparity in heterozygosity to be identified and the questionable chip data to be judged of unacceptable quality.

The simulation results (Figure 5 **)** show that genotype discordance can generate false associations, especially for genetic markers with low minor allele frequency. Thus, for genetic markers identified from GWAS with small odds ratios and small minor allele frequency values, our simulation suggests that more careful studies would be needed to confirm that genotyping errors did not result in the false associations. For example, SNP rs7578597 (in gene THADA), with a minor allele (C) frequency of 0.083, was identified as associated with Type 2 diabetes by a meta-analysis [42] that combined three GWAS data sets with an odds ratio of 1.25 (low bound of 95% confidence interval is 1.12 that is just slightly larger than the odds ratio that could be caused by genotyping errors alone, Figure 5B). However, no significant association for that SNP was observed in the three original GWAS [3], [4], [8]. Thus, it was not clear if the association was rendered true because of increased sample size in the meta-analysis, or is a false inference stemming from genotyping and other error sources. Careful replication studies were required to confirm the association [42].

In order to make simulations closely mimic an actual GWAS study, a case population of 2,000 samples and a control population of 3,000 samples were used; the size is very similar to that of the well-known Wellcome Trust Case Control Consortium studies [8]. Sample size has been increased to achieve higher statistical power in recent GWAS [20]–[26]. It is expected that the effect of an equivalent genotyping error rate would decrease in a GWAS when its sample size is increased. Therefore, simulations with a much larger sample size (5,000 cases and 5,000 controls) were conducted to examine the effect. Similar results (Figure S23 and Figure S24) were obtained though the odds ratios were decreased slightly, as expected.

## Supporting Information

Figure S1 **Layout of samples on the plates and corresponding information.**
(DOC)

Figure S2 **QC results of raw data from Affymetrix platform.** Blue bars are for samples from genotyping experiment E2, red bars are for samples from genotyping experiment E1, and cyan bars are for samples from genotyping experiment E3.
(DOC)

Figure S3 **QC results of raw data from Illumina platform.** Red circles are for samples from genotyping experiment E4 and black are for samples from genotyping experiment E5.
(DOC)

Figure S4 **Concordance of genotypes between technical replicates from genotyping experiment E1 by using Affy6 platform.** Each column and each row represent a technical replicate of a sample indicated by the sample codes at the x-axis and y-axis that are listed in Table 1.
(DOC)

Figure S5 **Concordance of genotypes between technical replicates from genotyping experiment E2 by using Affy6 platform.** Each column and each row represent a technical replicate of a sample indicated by the sample codes at the x-axis and y-axis that are listed in Table 1.
(DOC)

Figure S6 **Concordance of genotypes between technical replicates from genotyping experiment E3 by using Affy6 platform.** Each column and each row represent a technical replicate of a sample indicated by the sample codes at the x-axis and y-axis that are listed in Table 1.
(DOC)

Figure S7 **Concordance of genotypes between technical replicates from genotyping experiment E4 by using Illu1M platform.** Each column and each row represent a technical replicate of a sample indicated by the sample codes at the x-axis and y-axis that are listed in Table 1.
(DOC)

Figure S8 **Concordance of genotypes between technical replicates from genotyping experiment E5 by using Illu1M platform.** Each column and each row represent a technical replicate of a sample indicated by the sample codes at the x-axis and y-axis that are listed in Table 1.
(DOC)

Figure S9 **Concordance of genotypes between technical replicates from genotyping experiment E1 and experiment E2 by using Affy6 platform.** Each column and each row represent a technical replicate of a sample indicated by the sample codes at the x-axis (genotyping experiment E2) and y-axis (genotyping experiment E1) that are listed in Table 1.
(DOC)

Figure S10 **Concordance of genotypes between technical replicates from genotyping experiment E1 and experiment E3 by using Affy6 platform.** Each column and each row represent a technical replicate of a sample indicated by the sample codes at the x-axis (genotyping experiment E3) and y-axis (genotyping experiment E1) that are listed in Table 1.
(DOC)

Figure S11 **Concordance of genotypes between technical replicates from genotyping experiment E2 and experiment E3 by using Affy6 platform.** Each column and each row represent a technical replicate of a sample indicated by the sample codes at the x-axis (genotyping experiment E3) and y-axis (genotyping experiment E2) that are listed in Table 1.
(DOC)

Figure S12 **Concordance of genotypes between technical replicates from genotyping experiment E4 and experiment E5 by using Illu1M platform.** Each column and each row represent a technical replicate of a sample indicated by the sample codes at the x-axis (genotyping experiment E5) and y-axis (genotyping experiment E4) that are listed in Table 1.
(DOC)

Figure S13 **Concordance of genotypes between technical replicates from genotyping experiment E1 by using Affy6 platform and genotyping experiment E4 by using Illu1M platform.** Each column and each row represent a technical replicate of a sample indicated by the sample codes at the x-axis (genotyping experiment E4) and y-axis (genotyping experiment E1) that are listed in Table 1.
(DOC)

Figure S14 **Concordance of genotypes between technical replicates from genotyping experiment E1 by using Affy6 platform and experiment E5 by using Illu1M platform.** Each column and each row represent a technical replicate of a sample indicated by the sample codes at the x-axis (genotyping experiment E5) and y-axis (genotyping experiment E1) that are listed in Table 1.
(DOC)

Figure S15 **Concordance of genotypes between technical replicates from genotyping experiment E2 by using Affy6 platform and experiment E4 by using Illu1M platform.** Each column and each row represent a technical replicate of a sample indicated by the sample codes at the x-axis (genotyping experiment E4) and y-axis (genotyping experiment E2) that are listed in Table 1.
(DOC)

Figure S16 **Concordance of genotypes between technical replicates from genotyping experiment E2 by using Affy6 platform and experiment E5 by using Illu1M platform.** Each column and each row represent a technical replicate of a sample indicated by the sample codes at the x-axis (genotyping experiment E5) and y-axis (genotyping experiment E2) that are listed in Table 1.
(DOC)

Figure S17 **Concordance of genotypes between technical replicates from genotyping experiment E3 by using Affy6 platform and experiment E4 by using Illu1M platform.** Each column and each row represent a technical replicate of a sample indicated by the sample codes at the x-axis (genotyping experiment E4) and y-axis (genotyping experiment E3) that are listed in Table 1.
(DOC)

Figure S18 **Concordance of genotypes between technical replicates from genotyping experiment E3 by using Affy6 platform and experiment E5 by using Illu1M platform.** Each column and each row represent a technical replicate of a sample indicated by the sample codes at the x-axis (genotyping experiment E5) and y-axis (genotyping experiment E3) that are listed in Table 1.
(DOC)

Figure S19 **Successful genotype call rates for 24 NDA replicates of the six subjects are represented by bars (left y-axis) color coded by subject.** Red: HapMap NA10385; Blue: HapMap NA12249; Magenta: HapMap NA12248; Cyan: NCTR59; Yellow: NCTR8; Green: NCTR13. Heterozygote call rates (right y-axis) are plotted as solid circles and overlaid onto the corresponding bars.
(DOC)

Figure S20 **Successful genotype call rates for 24 NDA replicates of the six subjects are represented by bars (left y-axis) color coded by subject.** Red: HapMap NA10385; Black and Blue: HapMap NA12249; Magenta: HapMap NA12248; Cyan: NCTR59; Yellow: NCTR8; Green: NCTR13. Heterozygote call rates (right y-axis) are plotted as solid circles and overlaid onto the corresponding bars.
(DOC)

Figure S21 **Concordance of genotypes between technical replicates from genotyping experiment E1 by using Affy6 platform with the replicate of low quality of included.** Each column and each row represent a technical replicate of a sample indicated by the sample codes at the x-axis and y-axis that are listed in Table 1.
(DOC)

Figure S22 **Concordance of genotypes between technical replicates from genotyping experiment E2 by using Affy6 platform with the replicates of low quality of included.** Each column and each row represent a technical replicate of a sample indicated by the sample codes at the x-axis and y-axis that are listed in Table 1.
(DOC)

Figure S23 **Simulations results (Sample size = 10,000: case: 5,000; control: 5,000).**
(DOC)

Figure S24 **Simulations results (Sample size = 10,000: case: 5,000; control: 5,000).**
(DOC)

Figure S25 **Concordance of genotypes between technical replicates from genotyping experiments by using Affy6 platform.** For HapMap subject NA10835 (**A**), there are 13 rows and columns: the first four are from genotyping experiment E1; the second four are from genotyping experiment E2; the third four are from genotyping experiment E3; and the last one is from HapMap data. For HapMap subject NA12249 (**B**), there are 11 rows and columns: the first four are from genotyping experiment E1; the next two are from genotyping experiment E2; the next four to experiment E2 are from genotyping experiment E3; and the last one is from HapMap data. For HapMap subject NA12248 (**C**), there are 13 rows and columns: the first four are from genotyping experiment E1; the next three are from genotyping experiment E2; the next four to experiment E2 are from genotyping experiment E3; and the last one is from HapMap data.
(DOC)

Figure S26 **Concordance of genotypes between technical replicates from genotyping experiments by using Illu1M platform.** For each of the three HapMap subjects, there are nine rows and columns. The first four represent genotyping results from genotyping experiment E4, the second four are referred to genotyping results from genotyping experiment E5, and the last one is the HapMap data from Illumina.
(DOC)
