# Supplementary material for: Technical Reproducibility of Genotyping SNP Arrays Used in Genome-Wide Association Studies
Source: PLoS One. 2012 Sep 7;7(9):e44483. doi: 10.1371/journal.pone.0044483 (PMC3436888; doi:10.1371/journal.pone.0044483)
Supplement: Figure S1 — Layout of samples on the plates and corresponding information. (DOC) [file pone.0044483.s001.doc]

|  | **1** | **2** | **3** | **4** | **5** | **6** | **7** | **8** | **9** | **10** | **11** | **12** |
| --- | --- | --- | --- | --- | --- | --- | --- | --- | --- | --- | --- | --- |
| **A** | **S6** | **S3** | **S6** |  |  |  |  |  |  |  |  |  |
| **B** | **S2** | **S2** | **S3** |  |  |  |  |  |  |  |  |  |
| **C** | **S6** | **S1** | **S2** |  |  |  |  |  |  |  |  |  |
| **D** | **S4** | **S1** | **S5** |  |  |  |  |  |  |  |  |  |
| **E** | **S1** | **S3** | **S5** |  |  |  |  |  |  |  |  |  |
| **F** | **S6** | **S4** | **S4** |  |  |  |  |  |  |  |  |  |
| **G** | **S4** | **S2** | **S5** |  |  |  |  |  |  |  |  |  |
| **H** | **S5** | **S3** | **S1** |  |  |  |  |  |  |  |  |  |

**DNA Row Col Code Sample**

DNA1 A1 S6 1 N13

DNA2 B1 S2 2 N12248

DNA3 C1 S6 3 N13

DNA4 D1 S4 4 N59

DNA5 E1 S1 5 N12249

DNA6 F1 S6 6 N13

DNA7 G1 S4 7 N59

DNA8 H1 S5 8 N8

DNA9 A2 S3 9 N10835

DNA10 B2 S2 10 N12248

DNA11 C2 S1 11 N12249

DNA12 D2 S1 12 N12249

DNA13 E2 S3 13 N10835

DNA14 F2 S4 14 N59

DNA15 G2 S2 15 N12248

DNA16 H2 S3 16 N10835

DNA17 A3 S6 17 N13

DNA18 B3 S3 18 N10835

DNA19 C3 S2 19 N12248

DNA20 D3 S5 20 N8

DNA21 E3 S5 21 N8

DNA22 F3 S4 22 N59

DNA23 G3 S5 23 N8

DNA24 H3 S1 24 N12249
