# Supplementary figures and images for: Technical Reproducibility of Genotyping SNP Arrays Used in Genome-Wide Association Studies
Source: PLoS One. 2012 Sep 7;7(9):e44483. doi: 10.1371/journal.pone.0044483 (PMC3436888; doi:10.1371/journal.pone.0044483)

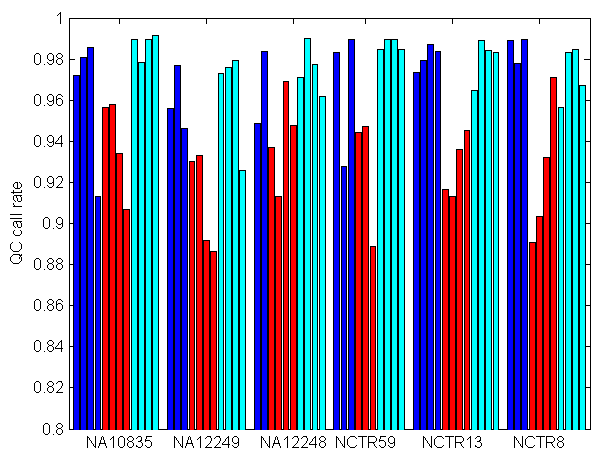

Supplement: Figure S2 — QC results of raw data from Affymetrix platform. Blue bars are for samples from genotyping experiment E2, red bars are for samples from genotyping experiment E1, and cyan bars are for samples from genotyping experiment E3. (DOC) [file pone.0044483.s002.doc]

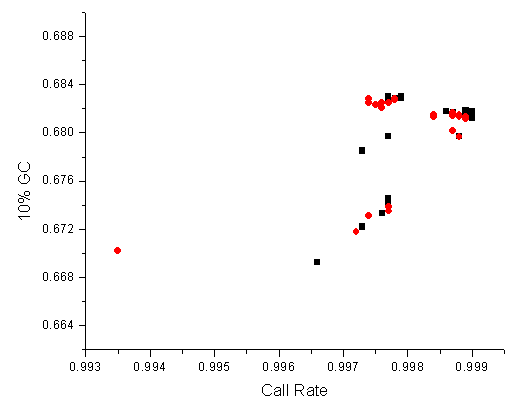

Supplement: Figure S3 — QC results of raw data from Illumina platform. Red circles are for samples from genotyping experiment E4 and black are for samples from genotyping experiment E5. (DOC) [file pone.0044483.s003.doc]

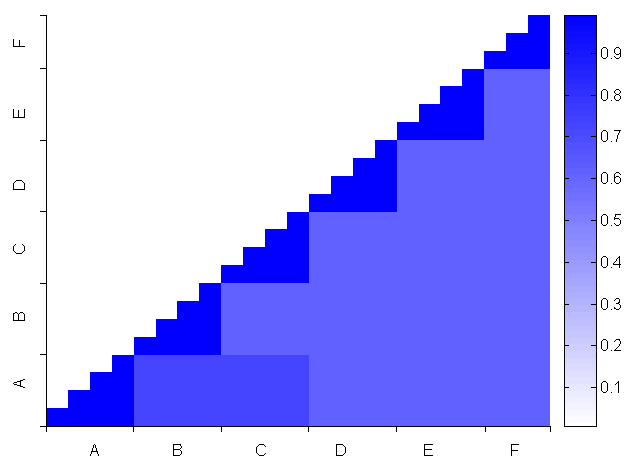

Supplement: Figure S4 — Concordance of genotypes between technical replicates from genotyping experiment E1 by using Affy6 platform. Each column and each row represent a technical replicate of a sample indicated by the sample codes at the x-axis and y-axis that are listed in Table 1. (DOC) [file pone.0044483.s004.doc]

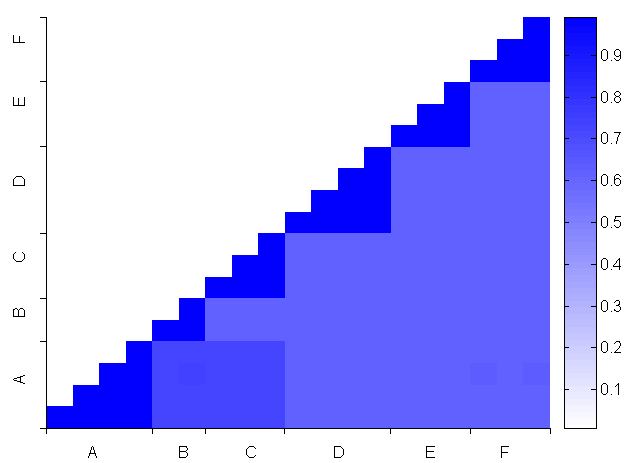

Supplement: Figure S5 — Concordance of genotypes between technical replicates from genotyping experiment E2 by using Affy6 platform. Each column and each row represent a technical replicate of a sample indicated by the sample codes at the x-axis and y-axis that are listed in Table 1. (DOC) [file pone.0044483.s005.doc]

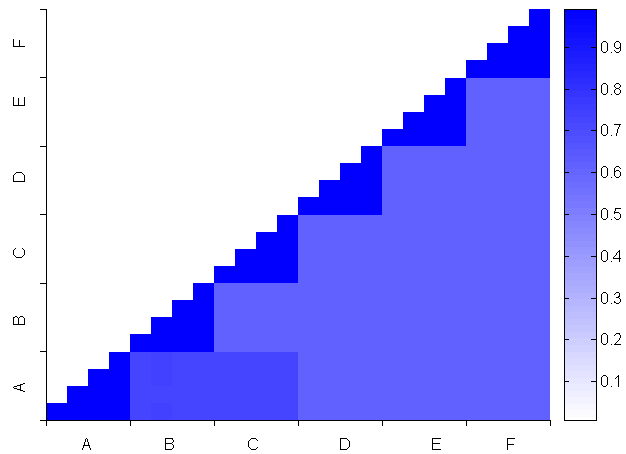

Supplement: Figure S6 — Concordance of genotypes between technical replicates from genotyping experiment E3 by using Affy6 platform. Each column and each row represent a technical replicate of a sample indicated by the sample codes at the x-axis and y-axis that are listed in Table 1. (DOC) [file pone.0044483.s006.doc]

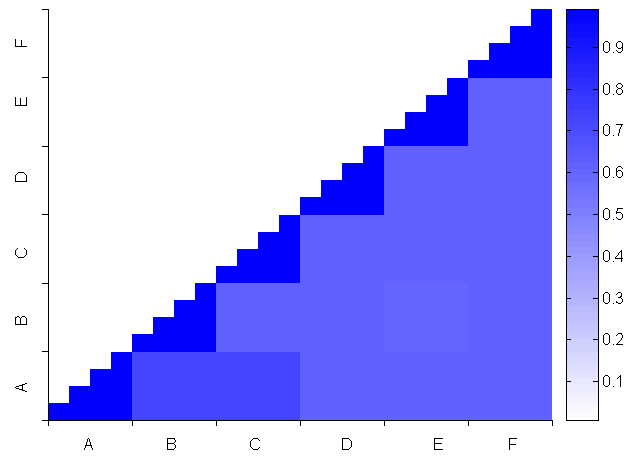

Supplement: Figure S7 — Concordance of genotypes between technical replicates from genotyping experiment E4 by using Illu1M platform. Each column and each row represent a technical replicate of a sample indicated by the sample codes at the x-axis and y-axis that are listed in Table 1. (DOC) [file pone.0044483.s007.doc]

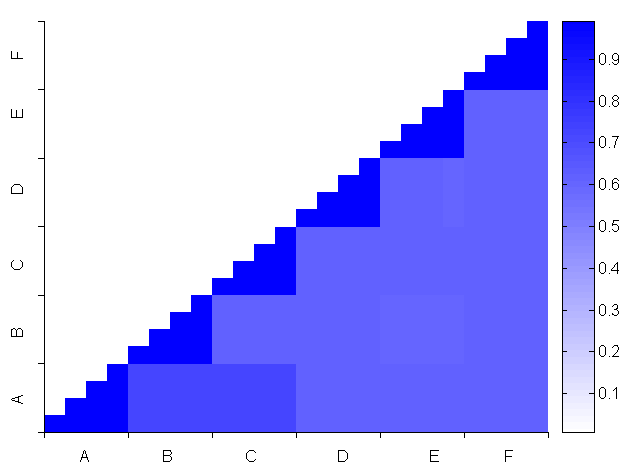

Supplement: Figure S8 — Concordance of genotypes between technical replicates from genotyping experiment E5 by using Illu1M platform. Each column and each row represent a technical replicate of a sample indicated by the sample codes at the x-axis and y-axis that are listed in Table 1. (DOC) [file pone.0044483.s008.doc]

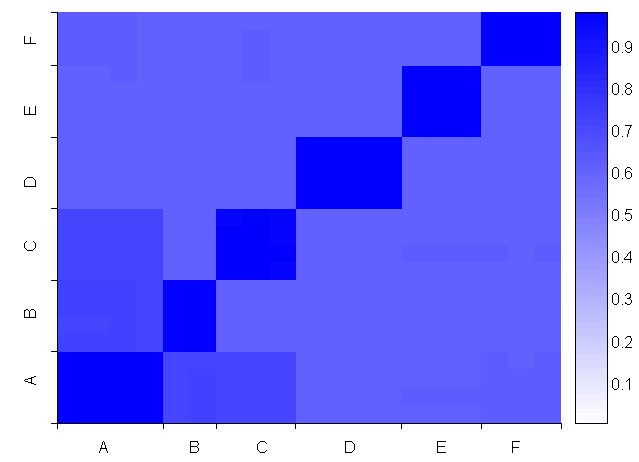

Supplement: Figure S9 — Concordance of genotypes between technical replicates from genotyping experiment E1 and experiment E2 by using Affy6 platform. Each column and each row represent a technical replicate of a sample indicated by the sample codes at the x-axis (genotyping experiment E2) and y-axis (genotyping experiment E1) that are listed in Table 1. (DOC) [file pone.0044483.s009.doc]

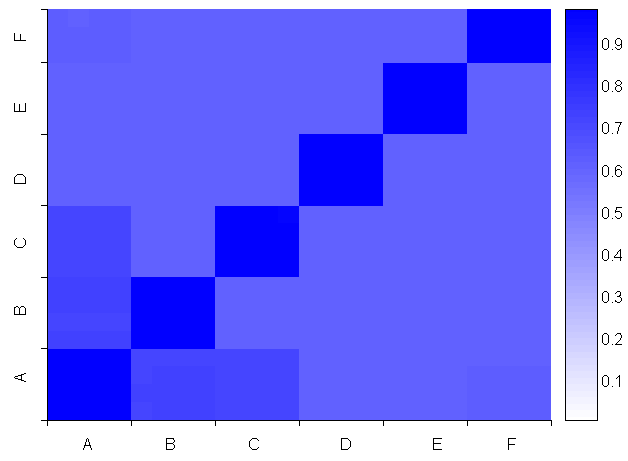

Supplement: Figure S10 — Concordance of genotypes between technical replicates from genotyping experiment E1 and experiment E3 by using Affy6 platform. Each column and each row represent a technical replicate of a sample indicated by the sample codes at the x-axis (genotyping experiment E3) and y-axis (genotyping experiment E1) that are listed in Table 1. (DOC) [file pone.0044483.s010.doc]

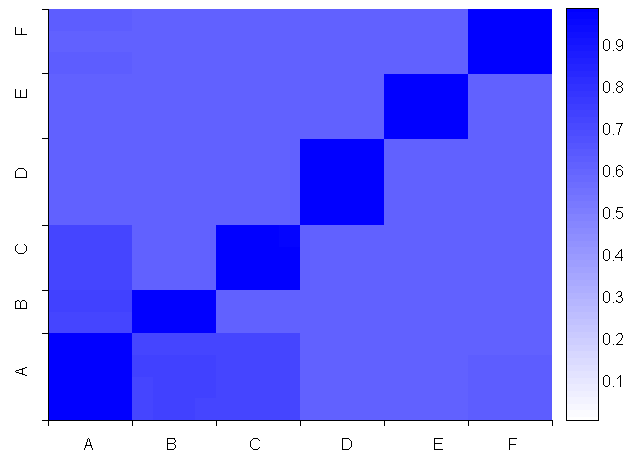

Supplement: Figure S11 — Concordance of genotypes between technical replicates from genotyping experiment E2 and experiment E3 by using Affy6 platform. Each column and each row represent a technical replicate of a sample indicated by the sample codes at the x-axis (genotyping experiment E3) and y-axis (genotyping experiment E2) that are listed in Table 1. (DOC) [file pone.0044483.s011.doc]

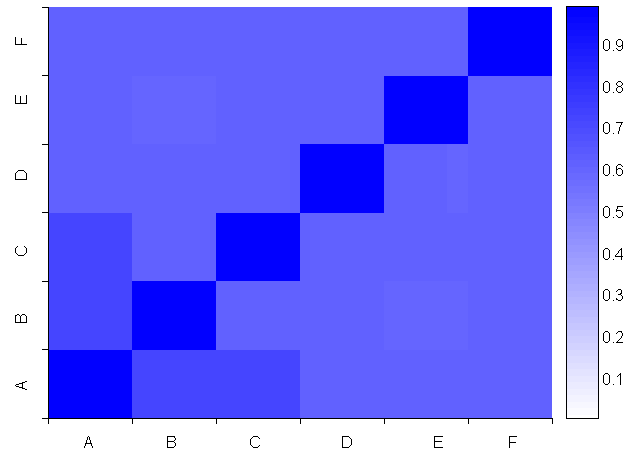

Supplement: Figure S12 — Concordance of genotypes between technical replicates from genotyping experiment E4 and experiment E5 by using Illu1M platform. Each column and each row represent a technical replicate of a sample indicated by the sample codes at the x-axis (genotyping experiment E5) and y-axis (genotyping experiment E4) that are listed in Table 1. (DOC) [file pone.0044483.s012.doc]

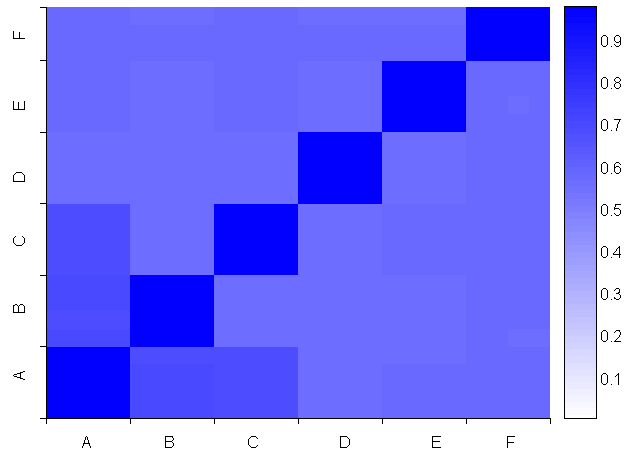

Supplement: Figure S13 — Concordance of genotypes between technical replicates from genotyping experiment E1 by using Affy6 platform and genotyping experiment E4 by using Illu1M platform. Each column and each row represent a technical replicate of a sample indicated by the sample codes at the x-axis (genotyping experiment E4) and y-axis (genotyping experiment E1) that are listed in Table 1. (DOC) [file pone.0044483.s013.doc]

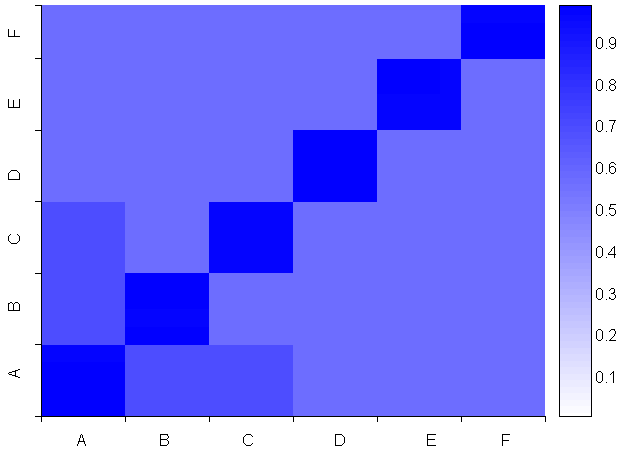

Supplement: Figure S14 — Concordance of genotypes between technical replicates from genotyping experiment E1 by using Affy6 platform and experiment E5 by using Illu1M platform. Each column and each row represent a technical replicate of a sample indicated by the sample codes at the x-axis (genotyping experiment E5) and y-axis (genotyping experiment E1) that are listed in Table 1. (DOC) [file pone.0044483.s014.doc]

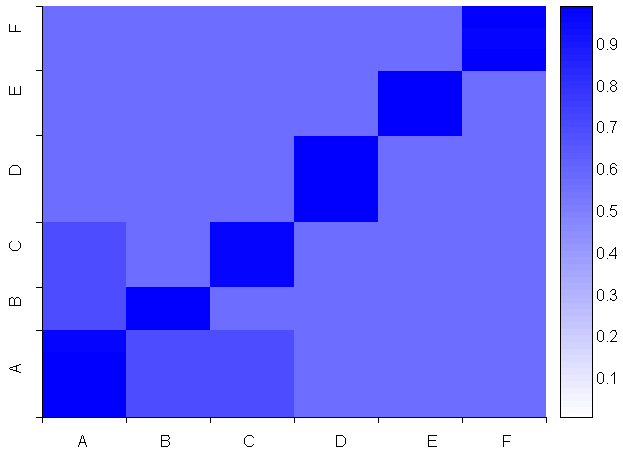

Supplement: Figure S15 — Concordance of genotypes between technical replicates from genotyping experiment E2 by using Affy6 platform and experiment E4 by using Illu1M platform. Each column and each row represent a technical replicate of a sample indicated by the sample codes at the x-axis (genotyping experiment E4) and y-axis (genotyping experiment E2) that are listed in Table 1. (DOC) [file pone.0044483.s015.doc]

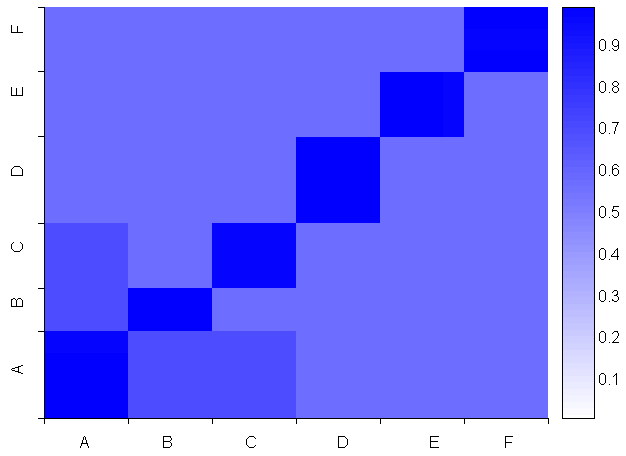

Supplement: Figure S16 — Concordance of genotypes between technical replicates from genotyping experiment E2 by using Affy6 platform and experiment E5 by using Illu1M platform. Each column and each row represent a technical replicate of a sample indicated by the sample codes at the x-axis (genotyping experiment E5) and y-axis (genotyping experiment E2) that are listed in Table 1. (DOC) [file pone.0044483.s016.doc]

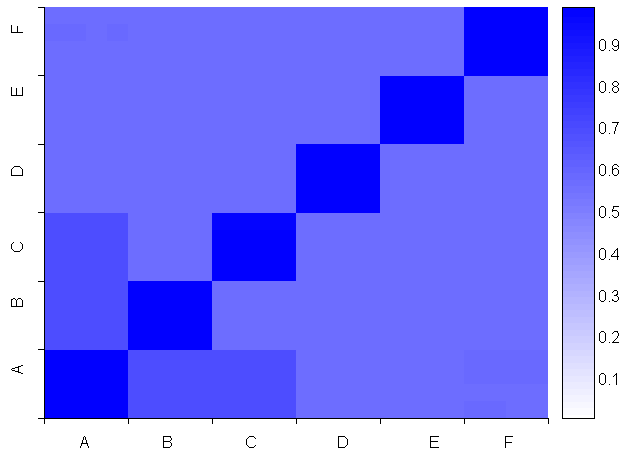

Supplement: Figure S17 — Concordance of genotypes between technical replicates from genotyping experiment E3 by using Affy6 platform and experiment E4 by using Illu1M platform. Each column and each row represent a technical replicate of a sample indicated by the sample codes at the x-axis (genotyping experiment E4) and y-axis (genotyping experiment E3) that are listed in Table 1. (DOC) [file pone.0044483.s017.doc]

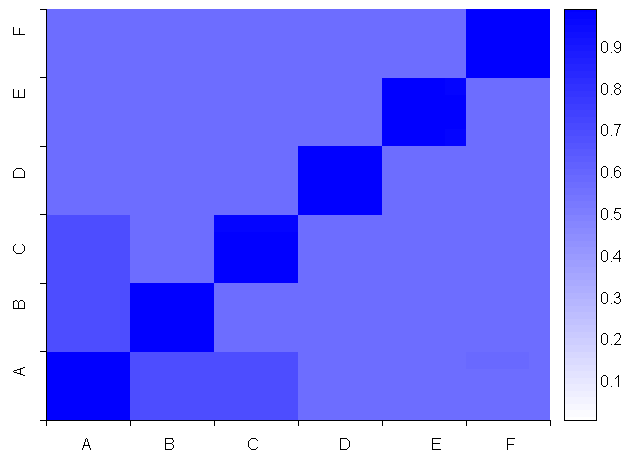

Supplement: Figure S18 — Concordance of genotypes between technical replicates from genotyping experiment E3 by using Affy6 platform and experiment E5 by using Illu1M platform. Each column and each row represent a technical replicate of a sample indicated by the sample codes at the x-axis (genotyping experiment E5) and y-axis (genotyping experiment E3) that are listed in Table 1. (DOC) [file pone.0044483.s018.doc]

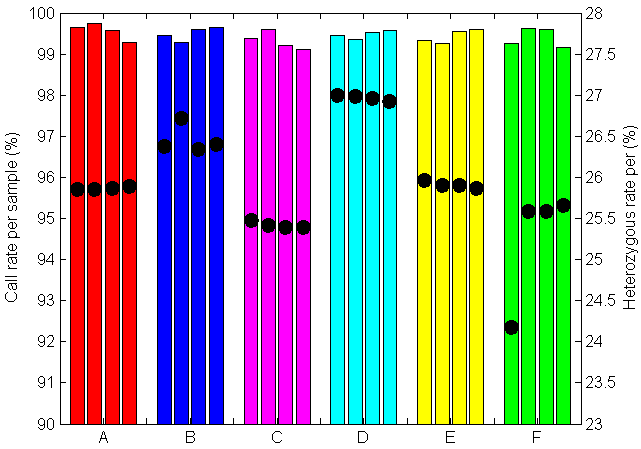

Supplement: Figure S19 — Successful genotype call rates for 24 NDA replicates of the six subjects are represented by bars (left y-axis) color coded by subject. Red: HapMap NA10385; Blue: HapMap NA12249; Magenta: HapMap NA12248; Cyan: NCTR59; Yellow: NCTR8; Green: NCTR13. Heterozygote call rates (right y-axis) are plotted as solid circles and overlaid onto the corresponding bars. (DOC) [file pone.0044483.s019.doc]

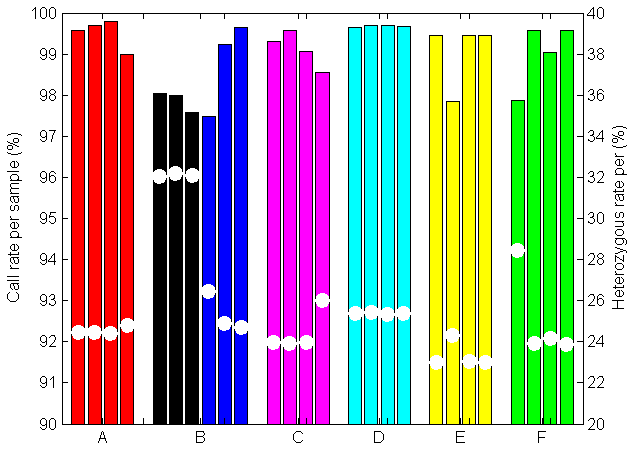

Supplement: Figure S20 — Successful genotype call rates for 24 NDA replicates of the six subjects are represented by bars (left y-axis) color coded by subject. Red: HapMap NA10385; Black and Blue: HapMap NA12249; Magenta: HapMap NA12248; Cyan: NCTR59; Yellow: NCTR8; Green: NCTR13. Heterozygote call rates (right y-axis) are plotted as solid circles and overlaid onto the corresponding bars. (DOC) [file pone.0044483.s020.doc]

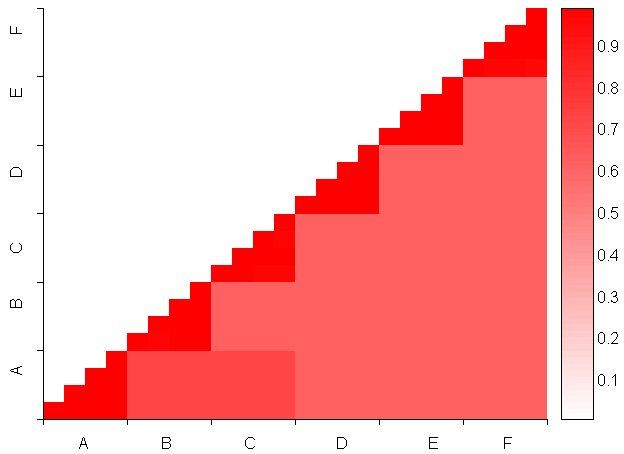

Supplement: Figure S21 — Concordance of genotypes between technical replicates from genotyping experiment E1 by using Affy6 platform with the replicate of low quality of included. Each column and each row represent a technical replicate of a sample indicated by the sample codes at the x-axis and y-axis that are listed in Table 1. (DOC) [file pone.0044483.s021.doc]

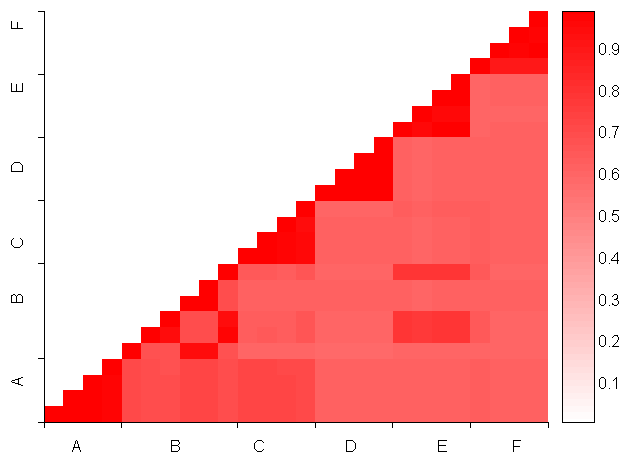

Supplement: Figure S22 — Concordance of genotypes between technical replicates from genotyping experiment E2 by using Affy6 platform with the replicates of low quality of included. Each column and each row represent a technical replicate of a sample indicated by the sample codes at the x-axis and y-axis that are listed in Table 1. (DOC) [file pone.0044483.s022.doc]

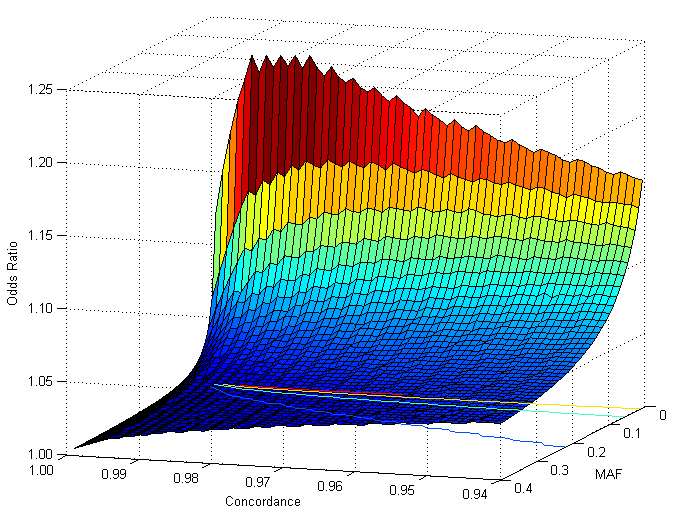

Supplement: Figure S23 — Simulations results (Sample size = 10,000: case: 5,000; control: 5,000). (DOC) [file pone.0044483.s023.doc]

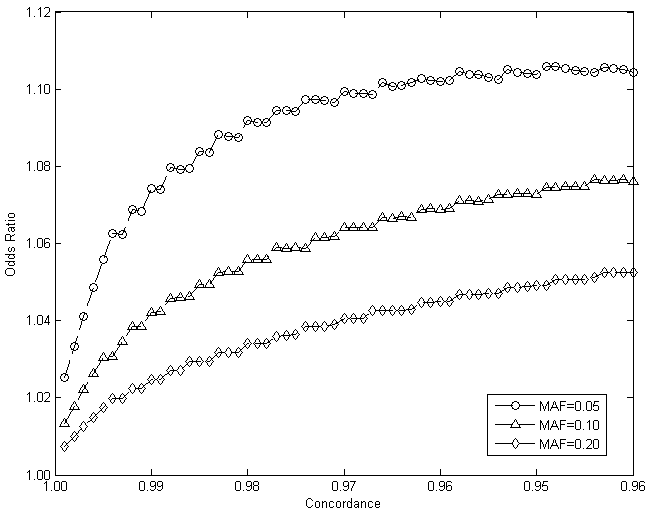

Supplement: Figure S24 — Simulations results (Sample size = 10,000: case: 5,000; control: 5,000). (DOC) [file pone.0044483.s024.doc]

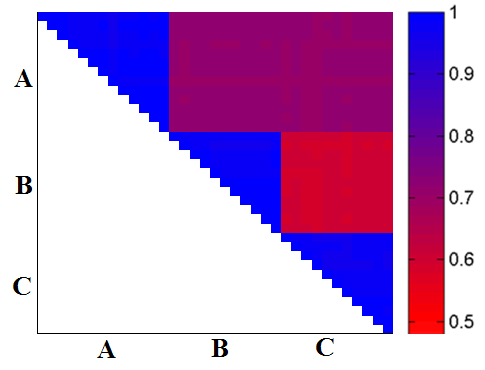

Supplement: Figure S25 — Concordance of genotypes between technical replicates from genotyping experiments by using Affy6 platform. For HapMap subject NA10835 (A), there are 13 rows and columns: the first four are from genotyping experiment E1; the second four are from genotyping experiment E2; the third four are from genotyping experiment E3; and the last one is from HapMap data. For HapMap subject NA12249 (B), there are 11 rows and columns: the first four are from genotyping experiment E1; the next two are from genotyping experiment E2; the next four to experiment E2 are from genotyping experiment E3; and the last one is from HapMap data. For HapMap subject NA12248 (C), there are 13 rows and columns: the first four are from genotyping experiment E1; the next three are from genotyping experiment E2; the next four to experiment E2 are from genotyping experiment E3; and the last one is from HapMap data. (DOC) [file pone.0044483.s025.doc]

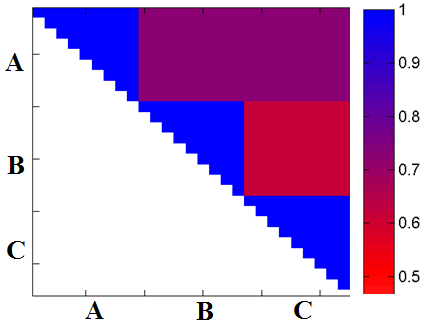

Supplement: Figure S26 — Concordance of genotypes between technical replicates from genotyping experiments by using Illu1M platform. For each of the three HapMap subjects, there are nine rows and columns. The first four represent genotyping results from genotyping experiment E4, the second four are referred to genotyping results from genotyping experiment E5, and the last one is the HapMap data from Illumina. (DOC) [file pone.0044483.s026.doc]
